# Blood SSR1: A Possible Biomarker for Early Prediction of Parkinson’s Disease

**DOI:** 10.3389/fnmol.2022.762544

**Published:** 2022-03-02

**Authors:** Wen Zhang, Jiabing Shen, Yuhui Wang, Kefu Cai, Qi Zhang, Maohong Cao

**Affiliations:** ^1^Department of Neurology, Affiliated Hospital of Nantong University, Nantong, China; ^2^Department of Microelectrics, Peking University, Peking, China; ^3^Key Laboratory of Neuroregeneration of Jiangsu and Ministry of Education, Co-innovation Center of Neuroregeneration, Nantong University, Nantong, China

**Keywords:** Parkinson’s disease, SSR1, biomarker, early diagnosis, artificial intelligence, machine learning

## Abstract

Parkinson’s disease (PD) is the second most common neurodegenerative disease associated with age. Early diagnosis of PD is key to preventing the loss of dopamine neurons. Peripheral-blood biomarkers have shown their value in recent years because of their easy access and long-term monitoring advantages. However, few peripheral-blood biomarkers have proven useful. This study aims to explore potential peripheral-blood biomarkers for the early diagnosis of PD. Three substantia nigra (SN) transcriptome datasets from the Gene Expression Omnibus (GEO) database were divided into a training cohort and a test cohort. We constructed a protein–protein interaction (PPI) network and a weighted gene co-expression network analysis (WGCNA) network, found their overlapping differentially expressed genes and studied them as the key genes. Analysis of the peripheral-blood transcriptome datasets of PD patients from GEO showed that three key genes were upregulated in PD over healthy participants. Analysis of the relationship between their expression and survival and analysis of their brain expression suggested that these key genes could become biomarkers. Then, animal models were studied to validate the expression of the key genes, and only SSR1 (the signal sequence receptor subunit1) was significantly upregulated in both animal models in peripheral blood. Correlation analysis and logistic regression analysis were used to analyze the correlation between brain dopaminergic neurons and SSR1 expression, and it was found that SSR1 expression was negatively correlated with dopaminergic neuron survival. The upregulation of SSR1 expression in peripheral blood was also found to precede the abnormal behavior of animals. In addition, the application of artificial intelligence technology further showed the value of SSR1 in clinical PD prediction. The three classifiers all showed that SSR1 had high predictability for PD. The classifier with the best prediction accuracy was selected through AUC and MCC to construct a prediction model. In short, this research not only provides potential biomarkers for the early diagnosis of PD but also establishes a possible artificial intelligence model for predicting PD.

## Introduction

Parkinson’s disease (PD) is a neurodegenerative disease principally defined by the motor symptoms of resting tremor, rigidity, and bradykinesia. These symptoms occur mainly because of the progressive loss of dopaminergic neurons in the substantia nigra pars compacta (SN; Damier et al., 1999; Kalia and Lang, 2015). However, the mechanism behind this neuronal loss remains largely unclear (Dauer and Przedborski, 2003). There is no cure for PD. The mainstay of its management is symptomatic treatment with drugs that increase dopamine concentrations or directly stimulate dopamine receptors (Kalia and Lang, 2015). Clinical diagnosis of PD is based on the presence of Parkinsonian motor features, but a significant proportion of nigral neurons are lost before the onset of motor symptoms (Lang and Lozano, 1998), meaning that clinical diagnosis is likely to occur too late for the administration of disease-modifying therapies. Therefore, in the management of PD, it is urgent to find reliable diagnostic and prognostic biomarkers of PD to prevent the loss of dopaminergic neurons at an early stage (Parnetti et al., 2019).

The latest biomarkers mainly detect α-synuclein (Visanji et al., 2014) and neuroimaging modalities (Brooks and Pavese, 2011). Cerebrospinal fluid (CSF) is close to the central nervous system, making it an ideal source of diagnostic markers for ongoing pathological processes. CSF α-synuclein appears to be reasonably sensitive and specific for PD (Hong et al., 2010; Mollenhauer et al., 2011). Total α-synuclein levels have been significantly decreased in PD patients compared with controls (Mollenhauer et al., 2011, 2013). However, obtaining CSF is difficult, and repeated lumbar puncture is not conducive to long-term monitoring. The detection of α-synuclein in plasma and serum remains controversial; some researchers found that it was unaffected in PD patients (Smith et al., 2012), while another study found that it was lower in them than controls (Besong-Agbo et al., 2013). Dopamine transporter imaging and magnetic resonance imaging of the SN are sensitive and specific tools for PD (Benamer et al., 2000; Kagi et al., 2010; Lehericy et al., 2012). Although these techniques are very sensitive, they are expensive and involve radiation exposure, and it is not known how useful they are for the early detection of atypical PD (Frosini et al., 2017). As blood is easier, cheaper, and less invasive to obtain than cerebrospinal fluid (Thambisetty and Lovestone, 2010), people have focused on biomarkers in blood (Chahine et al., 2014; Lin et al., 2019; Grossi et al., 2021), especially for longitudinal evaluation. Uric acid, miR-124, and other molecules can be used as biomarkers for the diagnosis of PD in peripheral blood (Angelopoulou et al., 2019; Lawton et al., 2020). However, a single biochemical marker is unlikely to be sufficient for the early diagnosis of PD, while a combination of them may be useful. Therefore, there is a need to find more PD biomarkers in peripheral blood, and the development of reliable and accurate peripheral-blood biomarkers will greatly promote the early detection of PD and the identification of its biological characteristics.

Massively parallel microarray analysis can reliably assess the relationships between gene expression and clinical manifestations on a global scale and reveal the etiology of complex diseases by identifying abnormalities in genes or pathways (Schadt et al., 2005). Weighted gene co-expression network analysis (WGCNA) and protein–protein interaction networks (PPI) were constructed here to identify hub genes underlying PD. Longitudinal studies over time are a common method for studying degenerative diseases. We established a time axis to explore the dynamic changes in hub gene expression in a PD model and their potential as biomarkers in the early stage of the model. Finally, machine learning is a key method of modern medical research, and it is often used to diagnose diseases or to screen biomarkers of them (Deo, 2015). In this study, we used random forest (RF), K-nearest neighbor (KNN) and support vector machine (SVM) to establish a PD prediction model (Zhang, 2016; Kriegeskorte and Golan, 2019). A previous study combined KNN with a genetic algorithm to achieve high classification accuracy (Zhang et al., 2018). Here, after comparing the AUC and MCC of three classifiers, an SVM was selected to build an artificial intelligence prediction model of PD in the early stage.

## Materials and Methods

### Gene Expression Data and Subsequent Processing Based on GEO Databases

**Figure 1:**
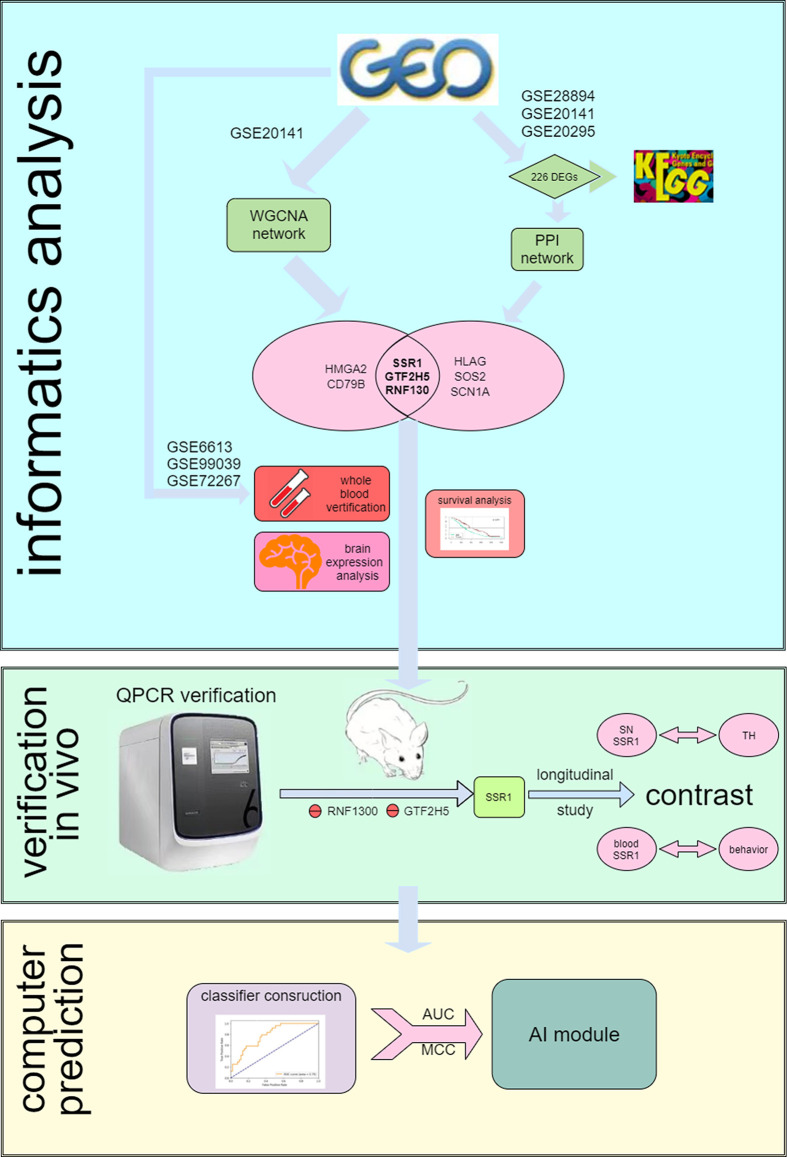
Flow chart of the analysis process.

The Gene Expression Omnibus (GEO^1^) is a public functional genomics data repository of high-throughput gene expression data, chips, and microarrays. As shown in the flow chart (Figure 1), we searched GEO with the following keywords: “(Parkinson’s disease) and (substantia nigra striatum)”, which yielded many datasets (Edgar et al., 2002). Four gene expression datasets [GSE28894, GSE20141, GSE20295, and GSE20292] were chosen and downloaded from GEO. The GSE28894 dataset contained 60 PD samples and 86 normal samples. GSE20141 contained 10 PD samples and eight normal samples. GSE20295 contained 40 PD samples and 53 normal samples. First, GSE20141 was chosen to run WGCNA to identify candidate hub genes. Second, GSE28894, GSE20141, and GSE20295 were used to construct a PPI network. GSE20292 was used to do external verification (Supplementary Figure 2). Then we searched for the keywords “(Parkinson’s disease) and (whole blood) and (early stage)” and obtained three datasets: GSE6613 GSE72267, and GSE99039. We performed whole blood verification of the hub genes in all the three datasets. GSE6613 was used to calculate the area under the receiver operating characteristic curve (AUC) of SSR1 and to build our machine learning model. We finally retrieved the datasets GSE85426, GSE51759, GSE89093, GSE138118, and GSE167914 for Alzheimer’s disease (AD), Huntington’s disease (HD), endometrial carcinoma, bladder cancer, and thyroid carcinoma, respectively, which were used to calculate the specificity of SSR1 to PD. Detailed of all data sets can be seen in Table 1.

**Table 1:** The information of Gene Expression Omnibus (GEO) datasets.

GEO datasets	Tissue	Disease	Application
GSE20141	SN	Parkinson’s disease	WGCNA analysis, PPI network
GSE28894	SN	Parkinson’s disease	PPI network
GSE20295	SN	Parkinson’s disease	PPI network
GSE6613	Whole blood	Parkinson’s disease	External verification machine learning
GSE72267	Whole blood	Parkinson’s disease	External verification
GSE99039	Whole blood	Parkinson’s disease	External verification
GSE85426	Whole blood	Alzheimer’s disease	machine learning (specificity of SSR1 to PD)
GSE51759	Whole blood	Huntington’s Disease	machine learning (specificity of SSR1 to PD)
GSE89093	Whole blood	Endometrial Carcinoma	machine learning (specificity of SSR1 to PD)
GSE138118	Whole blood	Bladder Cancer	machine learning (specificity of SSR1 to PD)
GSE167914	Whole blood	Thyroid Carcinoma	machine learning (specificity of SSR1 to PD)

### WGCNA

In WGCNA, the correlation between modules and clinical subtypes is calculated according to the feature vector of each network module. Module eigengenes actually formulate the expression patterns of all genes within a given module into a single characteristic expression profile. Module eigengenes can be regarded as the first principal component of the gene module. The correlation between each gene in these modules was quantified by the gene significance (GS) value. Accordingly, the module significance (MS) of a certain module is defined as the averaged GS values of all genes included in it. Modules are ranked according to the MS score, and the top five modules are considered key modules relevant to clinical outcomes for further analysis. Hub genes in the co-expression network are a class of genes that have high connectivity within a network module and are significantly correlated with biological function (Chen et al., 2017). In this study, we measured the absolute value of the gene significance (GS) score, which represents the correlation between the genes in these modules and each phenotype (Yang et al., 2018). We screened candidate genes using the cutoff criteria |MM| ≥ 0.8 and |GS| ≥ 0.5 because such genes are biologically meaningful. |MM| ≥ 0.8 indicates that the gene is strongly related to the module, and |GS| ≥ 0.5 requires that the gene expression profile be closely related to each module.

### PPI Network Construction and Module Analysis

The differentially expressed genes (DEGs) between PD and normal samples were screened using GEO2R^2^). GEO2R is an interactive web tool that allows users to compare two or more datasets in a GEO series to identify DEGs across experimental conditions (Edgar et al., 2002). The adjusted *P*-values (*P*_adj._) and Benjamini and Hochberg false discovery rates were applied to provide a balance between the discovery of statistically significant genes and the limitation of false positives. An absolute value of the logarithm of the fold change (logFC) >1 and *P*_adj._ < 0.01 were considered statistically significant.

The PPI network was predicted using the Search Tool for the Retrieval of Interacting Genes (STRING^3^) online database. Analyzing the functional interactions between proteins may provide insights into the mechanisms of the generation or development of diseases. The PPI network of DEGs was constructed using the STRING database, and an interaction with a combined score >0.4 was considered statistically significant. Cytoscape is an open source bioinformatics software platform for visualizing molecular interaction networks. The plug-in Molecular Complex Detection (MCODE) of Cytoscape is an app for clustering a given network based on its topology to find densely connected regions. The PPI networks were drawn using Cytoscape, and the most significant module in the PPI networks was identified using MCODE. The criteria for selection were as follows: MCODE scores >5, degree cutoff = 2, node score cutoff = 0.2, max depth = 100 and *k*-score = 2. The hub genes in the PPI network were those with degree ≥10.

### Functional Analysis of Hub Genes and Enrichment Analysis of DEGs

The overall survival and disease-free survival analyses of hub genes were performed using Kaplan-Meier curves in cBioPortal^4^. The expression levels of six hub genes in the brain were determined from the NCBI database. The Database for Annotation, Visualization, and Integrated Discovery (DAVID^5^) is an online biological information database that integrates biological data and analysis tools and provides a comprehensive set of functional annotation information on genes and proteins for users to extract biological information. The Kyoto Encyclopedia of Genes and Genomes (KEGG) is a database resource for understanding high-level functions and biological systems from large-scale molecular datasets generated by high-throughput experimental technologies. Gene Ontology (GO) is a major bioinformatics tool to annotate genes and analyze the biological processes of these genes. To analyze the functions of DEGs, biological analyses were performed using the DAVID online database. *P* < 0.01 was considered statistically significant.

### Classifier Construction and Machine Learning

Three ML algorithms, SVM (De Martino et al., 2008), kNN (Cover and Hart, 1967), and RF (Ho, 1998) were built both to verify if SSR1 can distinguish PD patients well and to determine which best classifies SSR1 in PD datasets. The RF method is a commonly-used classification method containing a number of decision trees. A final classification label was determined based on the class with the most votes from all trees. RF is easily parallelizable and can be enhanced with boosting or bagging. kNN performs classification by assigning a point to the class that is most prevalent out of the k points closest to it. At the same time, kNN is simple to implement and can utilize Multi-task learning. SVM maps each data item into an n-dimensional feature space where n is the number of features. It then identifies the hyperplane that separates the data items into two classes while maximizing the marginal distance for both classes and minimizing the classification errors. It is important to note that each technique has its own advantages and disadvantages. We hope to use different algorithms for verification with complementary advantages to more comprehensively verify the feasibility of SSR1 as a biomarker.

All models were learned from the same training data generated by selecting 80% of the data, and the remaining 20% were used as validation data to measure and compare the performance of the model. Each algorithm was also tested with combinations of parameters; finally, we found that *c* = 2 for the SVM, *k* = 4 for the kNN, 60 trees for RF produced the best results. To evaluate the overall performance of each model, a 10-fold cross validation was performed. Of the 10 divided sets from the data, the process by which the learned model predicts the remaining one set was repeated 10 times, and eventually, all data were used for validation. All ML algorithms were implemented in the python package *sklearn*.

### Performance Evaluation

In order to find out the best classifier for further study, the performance of data validation was calculated according to the area under the curve (AUC) from 0.5 to 1 and the Matthews Correlation Coefficient (MCC) from −1 to 1, a parameter able to reflect classifier effectiveness (Chicco and Jurman, 2020).


$$MCC=\frac{TP\times TN-FP\times FN}{\sqrt{\left(TP+FP\right)\left(TP+FN\right)\left(TN+FP\right)\left(TN+FN\right)}}$$

TP is the number of samples correctly predicted as PD in PD samples, FN is the number of samples incorrectly predicted as NORMAL in PD samples, FP is the number of samples incorrectly predicted as PD in normal samples, and TN is the number of samples correctly predicted as NORMAL in normal samples. MCC ranges from −1 to 1, with a completely wrong classification at −1 and perfect classification at 1.

MCC of classification is defined as:


$$MCC=\frac{c\times s-\sum_{k}^{K}p_{k}\times t_{k}}{\sqrt{\left(S^{2}-\sum_{k}^{K}p_{k}^{2}\right)\times }\left(S^{2}-\sum_{k}^{K}t_{k}^{2}\right)}$$

*s* is the total number of samples, *c* is the total number of correctly predicted samples, $t_{k}=\sum_{i}^{K}C_{ik}$ is the number of all samples in class *k*, and $p_{k}=\sum_{i}^{K}C_{ki}$ and is the number of correctly predicted samples in class *k*. MCC of pan-cancer classification for perfect prediction is 1, but the minimum is somewhere between −1 and 0, depending on the number and distribution of the actual labels (Kim et al., 2020). Eventually, the classifier with the greatest AUC and MCC value was identified as the optimal PD classifier.

### Animal Experiments

All experimental protocols were performed following the guidelines on animal research provided by the institutional ethics committee at Nantong University and were approved by the committee.

**6-OHDA** Lesion: Adult C57BL/6J male mice (25–30 g) were maintained under a 12-h light/12-h dark cycle in cages and acclimated to the experimental environment for 1 week before modeling. The mice received a unilateral intrastriatal injection of 6-OHDA (Sigma-Aldrich, St. Louis, MO, USA). The animals were pretreated with desipramine (Sigma-Aldrich, St. Louis, MO, USA). A total dose of 12 μg of 6-OHDA dissolved in 3 μl PBS (16 μmol/ml) was infused into the right striatum at the following coordinates: anterior-posterior (AP), +0.09 cm; medial-lateral (ML), +0.22 cm; dorsal-ventral (DV), −0.25 cm relative to the bregma.

**MPTP model**: In the same mice, MPTP (Sigma-Aldrich, St. Louis, MO, USA) was intraperitoneally injected four times at an individual dose of 12 mg/kg dissolved in 200 μl PBS with a 2-h interval between the injections. Te control animals received saline only.

**Behavioral** Testing: All the tests were performed 0 d, 1 d, 3 d, 5 d, 7 d, 14 d, and 28 d after 6-OHDA injection in comparison with the normal group. **In the pole test**, the mice were placed head-upward on top of a rough-surfaced iron pole (50 cm in length and 1.0 cm in diameter) and could climb down to the base of the pole. The time that it took for each mouse to turn completely downward and then reach the floor was measured, with a cutoff of 120 s. The average of three measurements was taken as the result. **In apomorphine-induced rotation**, the mice were allowed to habituate for 10 min in a white 30 × 30-cm chamber. After an intraperitoneal injection of 0.5 mg/kg apomorphine hydrochloride (Sigma-Aldrich, St. Louis, MO, USA), the full rotations in the chamber were recorded with a video camera for 30 min and counted by a blinded examiner.

**Tissue** Preparation: Perfusion was performed with a cold saline solution, and fixation was then performed with 4% paraformaldehyde in 0.1 M phosphate buffer. Each brain was dissected, postfixed overnight in buffered 4% paraformaldehyde at 4°C and stored in a 30% sucrose solution at 4°C until it sank. Frozen sectioning was performed on a freezing microtome (Leica, CM3050S) to generate 20-μm-thick coronal sections.

**Mouse Plasma Extraction**: The researcher grabbed the scruff of the mouse with the left thumb, index finger, and middle finger, and the little finger and ring finger fixed the tail. The skin of the eye that needed to be removed was lightly pressed to make the eyeball become congested and prominent. Surgical scissors were used to cut off the beard of the mouse to prevent blood from leaving the beard and causing hemolysis. The eyeball was grasped with tweezers and quickly removed, and the blood flowed from the eye socket into an Eppendorf tube, which was supplemented with a 1:9 ratio of the anticoagulant. The supernatant obtained after centrifugation at 3,000 rpm for 5–10 min was plasma.

### Immunohistochemistry

The prepared tissue sections were washed with PBS, permeabilized with 0.25% Triton X-100 for 10 min at RT, and treated with 10% goat serum blocking buffer for 2 h at RT. Tissue sections were costained with primary antibody against tyrosine hydroxylase (TH; 1:300, Abcam, UK) as a marker for dopaminergic neurons overnight at 4°C. After washing, indirect fluorescence by incubating sections at room temperature in the dark for 1 h with goat anti-rabbit IgG conjugated with Alexa Fluor 568 (1:1,000, Life Technologies). The coverslips were then washed with PBST and treated with an antifade mounting medium with Hoechst 33342. Images were obtained under a microscope (Zeiss LSM700, Carl Zeiss Microimaging GmbH, Jena, Germany). All photographs were taken using the same exposure time. For immunocytochemistry, six to nine fields (two to three fields × three independent samples) were selected randomly from each group, and for immunohistochemistry, three sections from each animal (three mice) were randomly selected.

### Western Blotting Analysis

The brain tissue was homogenized in RIPA lysis buffer (EpiZyme, China), protease inhibitor cocktail (MCE, USA), and phosphatase inhibitor cocktail I (MCE, USA) and then centrifuged at 1,600× g at 4°C for 20 min. The supernatant was collected, and the protein concentration was determined using a BCA Protein Assay Kit (Beyotime, China). An aliquot of the supernatant was diluted in SDS-PAGE Sample Loading Buffer 28 (Beyotime, China), and the proteins were separated in Omni-PAGE^TM^ HEPES-Tris Gels (EpiZyme, China) and transferred to a polyvinylidene difluoride membrane (Millipore, USA). The membrane was blocked for 1 h at RT in blocking buffer comprising TBS with 5% Difco^TM^ skim milk (Becton, Dickinson and 606 Company, USA) and 0.1% Tween 20. It was then incubated with the following primary antibodies overnight at 4°C: rabbit anti-GAPDH (Abcam, UK), and rabbit anti-TH (Abcam, UK). The membrane was washed in TBST and incubated with goat anti-rabbit IgG (H + L) and cross-adsorbed secondary antibody (conjugated to horseradish peroxidase; Thermo Fisher, USA) for 1 h at RT. The membrane was then washed three times in TBST for 5 min. The antigen–antibody peroxidase complex was detected using High-sig ECL Western Blotting Substrate (Tanon^TM^, China) according to the manufacturer’s instructions, and images were obtained using the Tanon^TM^ 5200CE Chemi-Image System. The intensity of each band was determined with ImageJ Fiji 1.53c.

### RNA Extraction and Quantitative Real-Time PCR

Total RNA of the SN was extracted using TRIzol reagent (Tiangen, Beijing, China). The total RNA of plasma was extracted using an EZ-press Serum/Plasma RNA Purification Kit (EZBioscience, Beijing, China). The RNA of 3 mice was filtered through a filter column. Reverse transcription of the RNA into cDNA and quantitative polymerase chain reaction (qPCR) were performed according to the instructions of the PrimeScript RT Reagent Kit with gDNA Eraser (Takara, Dalian, China) and TB Green Premix Ex *Taq* II (Takara). Relative expression levels were obtained by normalizing glyceraldehyde phosphate dehydrogenase (GAPDH). Each reaction was performed in triplicate. The relative mRNA expression level was calculated by the comparative 2^−ΔΔCt^ method.

### Statistical Analysis

All data are presented as the means ± SEM and were analyzed using GraphPad Prism 8.0. The difference between two groups was analyzed by a two-tailed Student’s *t*-test, and one-way ANOVA followed by Tukey’s *post hoc* analysis was used for multiple comparisons among two or more groups. Significant difference among groups was assessed as ns *p* > 0.05, *p < 0.05, **p < 0.01, and ***p < 0.001.

## Results

### Determination of Hub Modules and Genes in WGCNA

**Figure 2:**
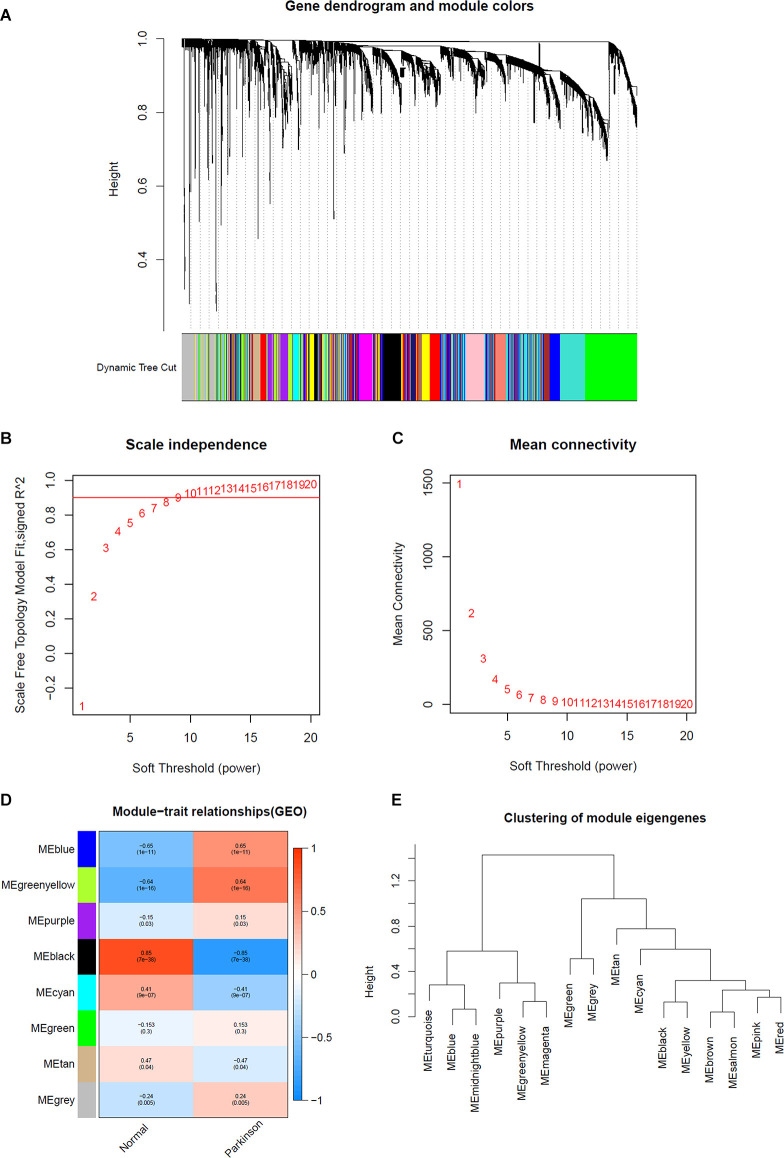
Determination of soft-thresholding power in WGCNA analysis. **(A)** Dendrogram of all differentially expressed genes clustered based on a dissimilarity measure. **(B)** Analysis of the scale-free fit index for various soft-thresholding powers β. **(C)** Analysis of the mean connectivity for various soft thresholding powers. **(D)** Heatmap of the correlation between module eigengenes and clinical traits of Parkinson. **(E)** Clustering of module eigengenes.

The expression profiles of several modules are included in Figure 2E, and each gene was classified into different modules (Figure 2A). We processed the gene expression profiles using variance analysis on the GSE20141 dataset, which included the most genes. The top five gene modules were used to select the hub gene module. To ensure that the network was a scale-free network, we ran an empirical analysis to choose an optimal parameter β. Both the scale-free topology model fit index and mean connectivity reached the steady state when β was equal to 4 (Figures 2B,C). A total of five gene modules were identified *via* average link age hierarchical clustering, and each module is represented in different colors. We drew a heat map to explore the correlations between module eigengenes and clinical traits (Figure 2D). Each column in Figure 2D displays the correlation and corresponding *p*-value: the darker the color, the stronger the correlation coefficient. We found that five module eigengenes had the highest correlations. Scatter plots of the degree and *P*-value of Cox regression in the five modules are shown in the Supplementary Figure. Accordingly, we selected the genes that had cutoff criteria |MM| ≥ 0.8 and |GS| ≥ 0.5, which are SSR1, RNF130, GTF2H5, HMGA2, and CD79B. WGCNA can reflect the continuity of potential co-expression information and avoid information loss by setting artificial threshold parameters (Langfelder and Horvath, 2008). However, WGCNA only focuses on a single dataset, so it lacks universality. To make up for this, we also performed a PPI network analysis.

### PPI Network Analysis and Hub Gene Selection

**Figure 3:**
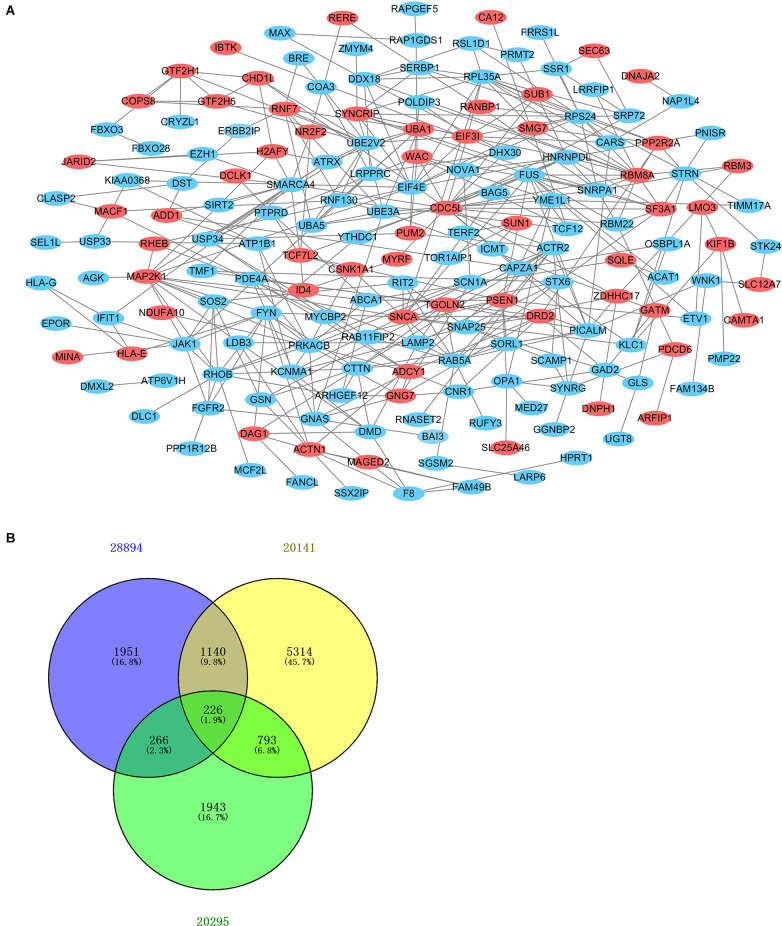
Venn diagram and PPI network. **(A)** The PPI network of DEGs was constructed using Cytoscape. Upregulated genes are marked in light red; downregulated genes are marked in light blue. **(B)** DEGs were selected with the absolute value of fold change >1 and *P*-value <0.01 among the mRNA expression profiling sets GSE28894, GSE20141, and GSE20295. The three datasets showed an overlap of 226 genes.

**Figure 4:**
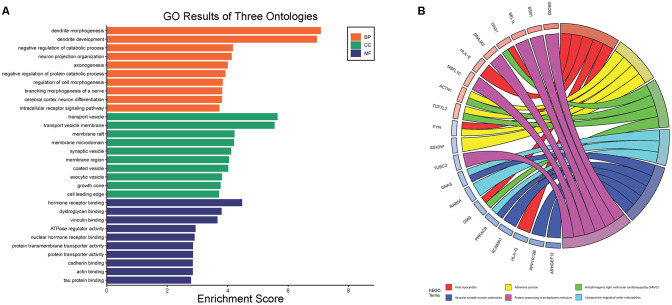
The Go terms and KEGG pathways enrichment analysis of 226 DEGs in PD. **(A)** The Go terms conclude the biological process, cellular component, and molecular function. **(B)** KEGG pathway revealed that DEGs were remarkably concentrated in the viral myocarditis, adherens junction, arrhythmogenic right ventricular cardlomyopathy (ARVC), vasopressin-regulated water reabsorption, vascular smooth muscle contraction, and protein processing in endoplasmic reticulum.

After standardization of the microarray results, DEGs were identified. The overlap between the three datasets contained 226 genes, as shown in the Venn diagram (Figure 3B), consisting of 154 downregulated genes and 72 upregulated genes in PD patients vs. healthy controls. We performed KEGG and GO analysis on the 226 genes and listed the top eight pathways in both analyses (Figure 4). GO function annotation results displayed that changes at the biological process (BP) were observably focused in dendrite morphogenesis, dendrite development, negative regulation of catabolic process, neuron projection organization, axonogenesis, and negative regulation of protein catabolic process (Figure 4A). Changes of DEGs significantly in cell component (CC) were mostly in transport vesicle, transport vesicle membrane, membrane raft, membrane microdomain, synaptic vesicle, and membrane region. The most enriched molecular function (MF) annotations were hormone receptor binding, dystroglycan binding, vinculin binding, ATPase regulator activity, nuclear hormone receptor binding, and protein transmembrane transporter activity. In addition, the results of the KEGG pathway analysis in the bubble chart revealed that DEGs were remarkably concentrated in the Viral myocarditis, Adherens junction, Arrhythmogenic right ventricular cardiomyopathy (ARVC), Vasopressin-regulated water reabsorption, Vascular smooth muscle contraction, and protein processing in the endoplasmic reticulum (Figure 4B). The pathways of hub genes were further investigated to determine the mechanism by which hub genes can act as biomarkers of PD. The PPI network of DEGs was constructed, and the most significant module was obtained using Cytoscape (Figure 3A). The results showed that the network contained six hub genes. These genes were identified as hub genes by virtue of having a degree ≥10. The genes shared in common by the WGCNA and PPI analysis were SSR1, GTF2H5, and RNF130. Since these genes were identified by two analytical methods, they will be the most reliable and representative of genes for our purposes. The names, abbreviations and functions of these hub genes are listed in Table 2.

**Table 2:** Three hub genes and functions.

No.	Gene symbol	Full name	Function
1	RNF130	ring finger protein 130	The protein encoded by this gene contains a RING finger motif and is similar to g1, a Drosophila zinc-finger protein that is expressed in mesoderm and involved in embryonic development. This gene may regulate growth factor withdrawal-induced apoptosis of myeloid precursor cells.
2	SSR1	signal sequence receptor subunit 1	The signal sequence receptor (SSR) is a glycosylated endoplasmic reticulum (ER) membrane receptor associated with protein translocation across the ER membrane. This gene generates several mRNA species as a result of complex alternative polyadenylation.
3	GTF2H5	general transcription factor IIH subunit 5	This gene encodes a subunit of transcript!on/repair factor TFIIH, which functions in gene transcription and DNA repair. This protein stimulates ERCC3/XPB ATPase activity to trigger DNA opening during DNA repair, and is implicated in regulating cellular levels of TFIIH.

### Whole-Blood Sample Verification and Hub Gene Analysis

**Figure 5:**
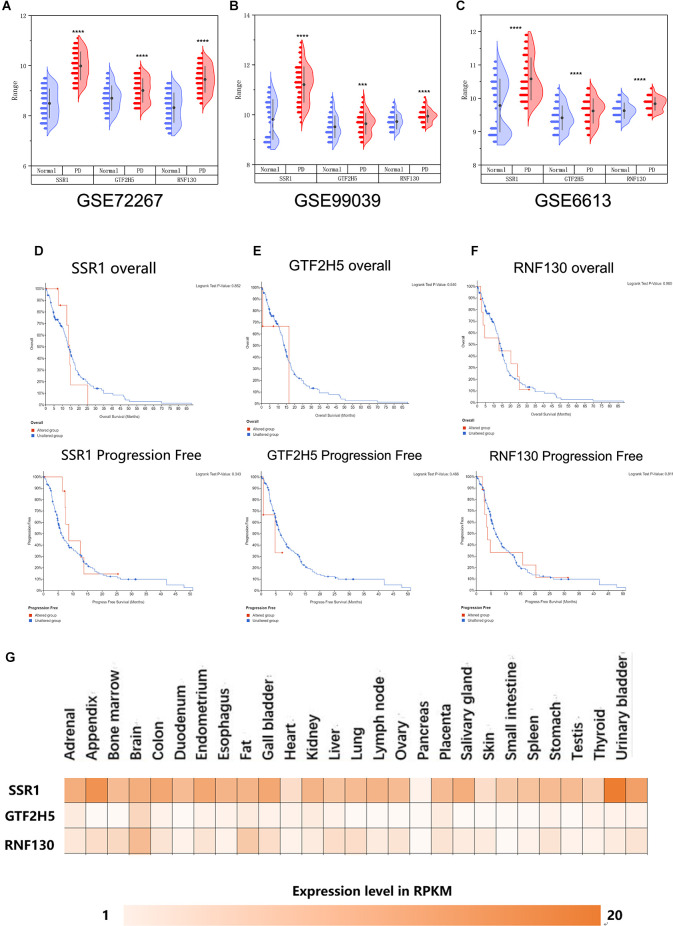
Analysis of the correlation between three hub genes and PD based on bioinfarmatics. **(A–C)** Verification of hub genes based on peripheral blood datasets: GSE72267, GSE99039, and GSE6613. **(D–F)** Overall survival and disease-free survival analyses of three hub genes were performed using cBioPortal online platform. *P* < 0.05 was considered statistically significant. **(G)** Expression level of three hub genes in brain.

To further explore whether the abnormally expressed hub genes in the brain could be detected in peripheral blood in patients at an early stage (at the onset of motor symptoms), we observed the difference in expression between the normal group and PD group in three whole-blood datasets and found that all three genes showed significantly upregulated in peripheral blood (Figures 5A–C). Their differential expression in peripheral blood was basically consistent with that in the brain. The overall survival analysis of the hub genes was performed using Kaplan-Meier curves. PD patients whose period blood highly expressed these genes showed good overall survival and disease-free survival (Figures 5D–F). SSR1, GTF2H5, and RNF130 were expressed highly in brain tissue (Figure 5G), which means they meet the fundamental requirements of biomarkers of PD.

### Expression Levels of Hub Genes *In vivo*

**Figure 6:**
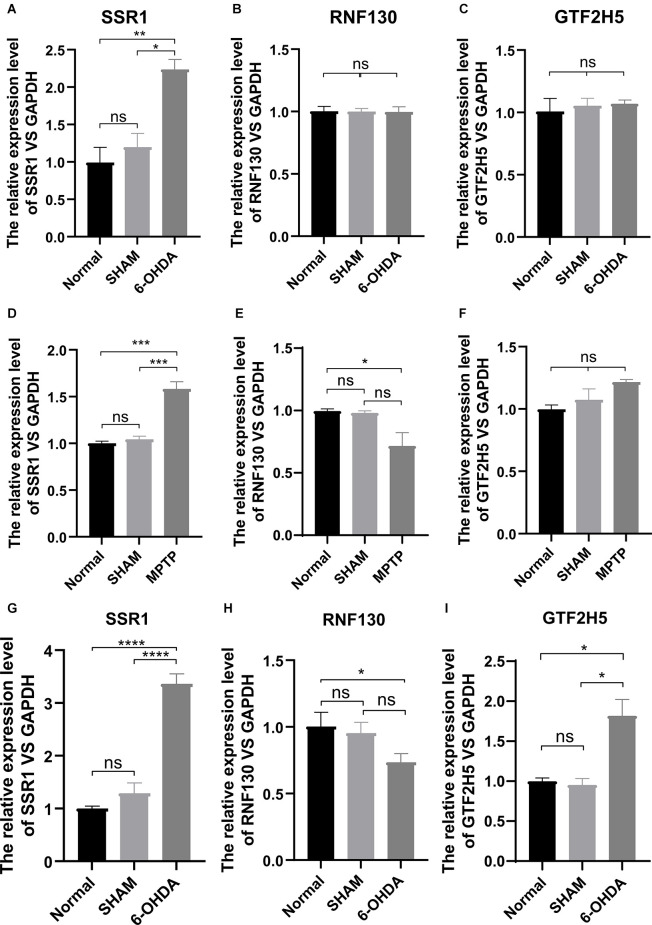
The mRNA relative expression levels of SSR1, GTF2H5, and RNF130 in PD model mice. **(A–C)** The expression levels of SSR1, GTF2H5, and RNF130 in SN (substantia nigra) *in vivo* in PD animal model constructed with 6-OHDA. **(D–F)** The expression levels of SSR1, GTF2H5, and RNF130 in SN (substantia nigra) *in vivo* in PD animal model constructed with MPTP. **(G–I)** The expression levels of SSR1, GTF2H5, and RNF130 in whole blood *in vivo* in PD animal model constructed with 6-OHDA. Norrnal group (non injected mice), SHAM group (PBS injected mice); ns *p* > 0.05, *p < 0.05, **P < 0.01, ***P < 0.001, ****P < 0.0001 vs. each group.

To analyze the accuracy and reliability of the above bioinformatic analysis, Quantitative Real-Time PCR was used to detect the expression levels of the hub genes in the SN and period blood of PD model mice. We used 6-OHDA and MPTP models for tissue verification. Compared with the value in normal SN tissue (non injected mice) and SHAM group, the expression level of SSR1 was significantly upregulated (*P* < 0.05) after 6-OHDA, as well as after MPTP injury (Figures 6A,D). GTF2H5 showed no significant difference in the 6-OHDA model and MPTP model (Figures 6C,F). RNF130 was not different in either model (Figures 6B,E). Considering the results above, we chose the 6-OHDA model to detect blood changes in hub genes. Surprisingly, SSR1 and GTF2H5 were both upregulated to varying degrees (Figures 6G–I). However, because they showed no obvious change in brain tissue, we thought that the changes in peripheral blood of GTF2H5 might not be directly related to PD. The imbalance of SSR1 both in the tissues and in peripheral blood after injury suggested that it may play an important role in the occurrence and progression of PD.

### Longitudinal Study of SSR1 Expression *In vivo*

**Figure 7:**
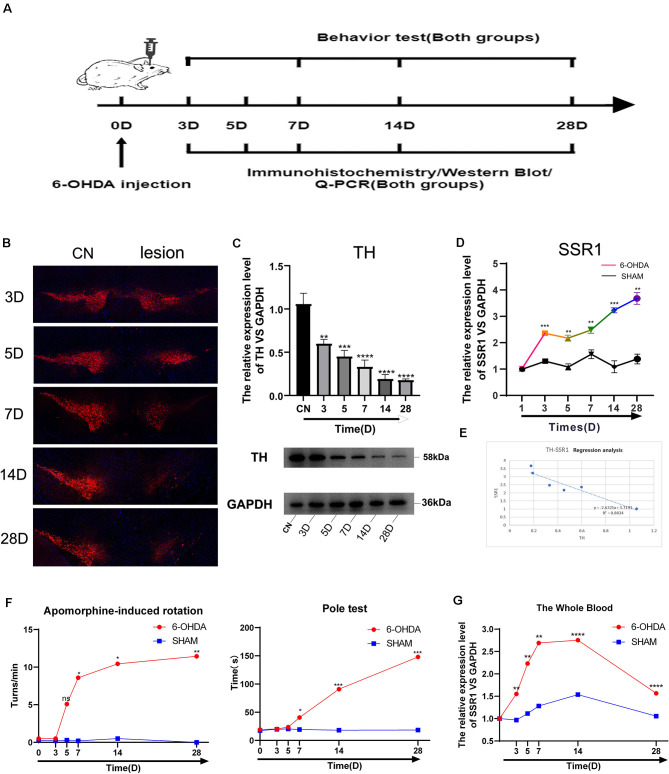
Longitudinal study of SSR1 expression *in vivo*. **(A)** The flowchart of the construction of the 6-OHDA subacute model, behavioral tests, and sacrifice. **(B)** Tyrosine hydroxylase (TH) staining of the substantia nigra (SN) of the above mice. Scale bars: 200 μm. **(C)** Western blot analyses of TH in SN of the above mice. **(D)** The mRNA relative expression levels of SSR1 in SN of the above mice. **(E)** The correlation analysis of SSR1 and TH in SN. **(F)** Pole tests and apomorphine-induced rotation were conducted by a blinded observer after 6-OHDA treatment. **(G)** The mRNA relative expression levels of SSR1 in the whole blood of the above mice. ns *p* > 0.05, *P < 0.05, **P < 0.01, ***P < 0.001, ****P < 0.0001 vs. Control group.

To further explore the relationship between the hub gene SSR1 and dopaminergic neurons in the SN, we established a time axis of 0, 3, 5, 7, 14, and 28 d (Figure 7A). We detected TH and SSR1 in the SN tissue of 6-OHDA-injured mice and with PBS-injected mice. We used Western blot and immunohistochemistry to analyze the change in TH. Immunohistochemical fluorescence showed that on day 7 after injury, dopaminergic neuron number began to decrease significantly. In the following days, the number of dopamine neurons remained low (Figure 7B). Western blot showed similar results: TH decreased below 60% of the control level at day 7, and from day 14 to day 28, it was lower than 20% (Figure 7C). Using qPCR to detect the expression trend of SSR1 at the same time, we found that the increase in SSR1 was divided into three stages (Figure 7D). It increased significantly from 0 d to 3 d, remained stable from 3 d to 7 d, and increased again from 7 d to 28 d, which was consistent with the decreasing trend of dopamine neurons. Then we performed a correlation analysis of SSR1 and TH in SN (Figure 7E). Regression of TH neuron number on SSR1 concentration showed a negative correlation, with a goodness of fit (R^2^) of 0.8834. The results show that in animal models, SSR1 has a strong negative correlation with TH neurons. SSR1 may be related to damage to TH neurons and to a certain extent can reflect the degree of damage to them.

To explore whether SSR1 could be a biomarker in the early stage of PD, we measured the correlation between changes in animal behavior and SSR1 expression in blood. Most preclinical experiments have focused on late-stage, chronic, fully DA-depleted states (Stanic et al., 2003; Grealish et al., 2010; Boix et al., 2015; Zhang et al., 2017). Few studies have focused specifically on the early-phase behavioral responses after 6-OHDA lesions in the SNc (Fornaguera and Schwarting, 1999; Rosa et al., 2020). In the 6-OHDA model, few researchers have focused on behavioral disorders in the first week after SN striatum injury. We thought it would be interesting to study the early time course of changes occurring in the emergence of the parkinsonian lesion in the standard 6-OHDA model and whether SSR1 might be predictive of the severity of the lesion. Behavioral changes began to appear at 7 days after 6-OHDA injection, and significant differences appeared from 14 days to 28 days. There were few abnormalities in 3D and 5D (in apomorphine-induced rotation, when the number of rotations is >7 r/min, it is considered a successful model; Figure 7F). The rotation experiment induced by apomorphine further suggested that the number of dopamine neurons decreased to less than 20% of the control level at 14D-28D. The expression of SSR1 in peripheral blood began to be upregulated as early as day 3, when behavioral disorders were not obvious (Figure 7G). As the course of the disease progressed, the expression of SSR1 in peripheral blood stayed high. These results show that in the early stage of a PD model (with few or no behavioral abnormalities), SSR1 is significantly upregulated in both the brain and blood. This abnormal expression may indicate the degree of damage to dopaminergic neurons and make SSR1 a promising biomarker of early PD.

### Machine Learning

**Figure 8:**
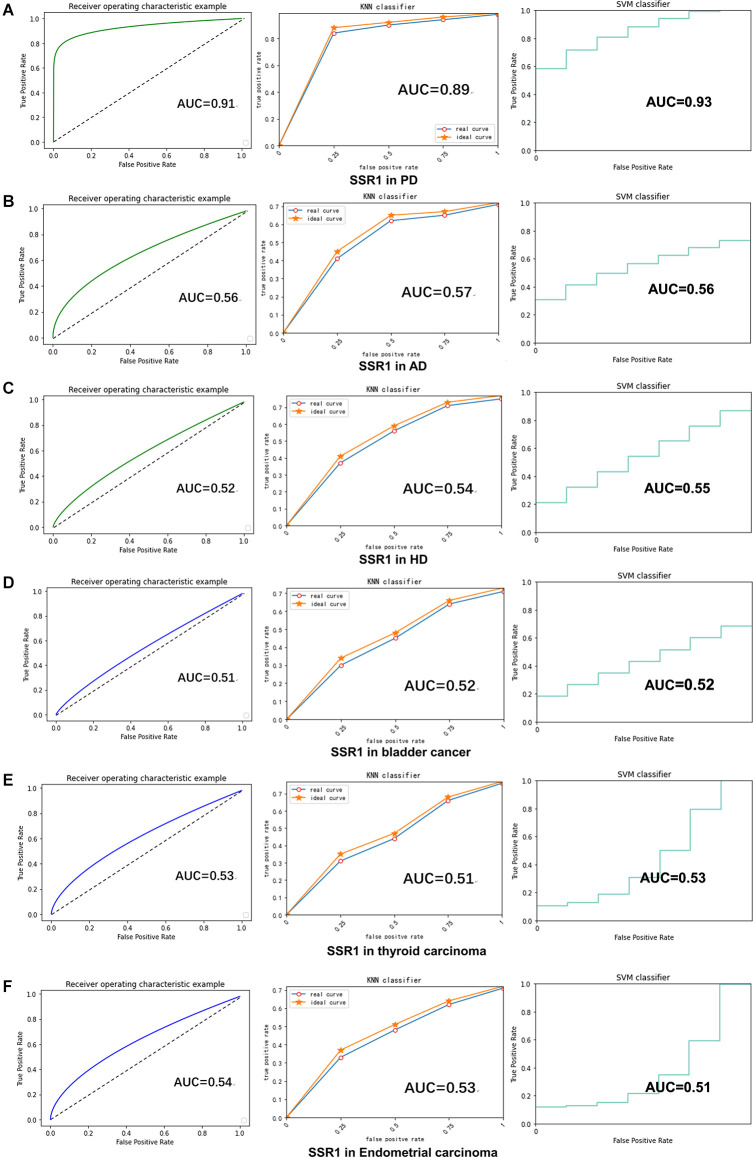
SSR1 in artificial intelligence prediction model. **(A)** The ROC curve of the sensitivity for the diagnosis of PD based on SSR1 from RF (left), KNN analysis (middle), and SVM analysis (right). **(B–F)** The ROC curve of the specificity for the diagnosis of PD based on SSR1 in AD **(B)**, HD **(C)**, Bladder cancer **(D)**, Thyroid carcinoma **(E)**, and Endometrial carcinoma **(F)**.

**Figure 9:**
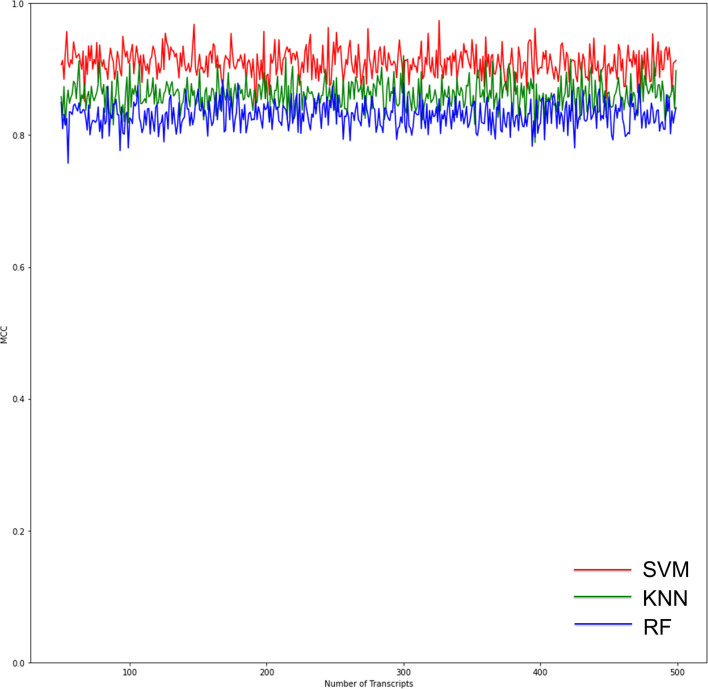
Graphs show the performance of the three ML models according to the number of genes for binary classification. The red curves indicate SVM classifiers. The green curves indicate KNN classifiers. The blue curves indicate RF classifiers. The SVM classifier had the highest Matthews correlation coefficient (MCC) value, which means the highest recognition accuracy and precision.

RF, KNN, and SVM were used to construct classifiers to distinguish PD patients from healthy controls based on GSE6613, which shows the best performance in both classifiers (Supplementary Figure 1). To identify the best predictors of each classifier, we added these upregulated genes to each classifier one by one in order of rank. The RF classifier based on SSR1 had good predictive power (AUC: 0.91; Figure 8A). In addition, we validated the PD specificity of our gene expression classifier by testing it on two different protein aggregation disease datasets: one Alzheimer’s disease dataset (GSE85426) and one Huntington’s disease expression dataset (GSE51799). The AUCs of SSR1 for AD and HD were low (Figures 8B,C), which indicates that our expression classifier has no prediction power for Alzheimer’s disease or Huntington’s disease but is efficient and specific for PD. Given that the expression level of SSR1 in other organs (Figure 6B), such as the bladder, thyroid, and endometrium, was similar to that in the brain, we chose three datasets of these diseases and tested the AUC power of SSR1 in cases not specific to PD. As expected, these curves had AUCs lower than 0.6 (Figures 8D–F), which means that SSR1 has extreme specificity to Parkinson’s disease, while it behaves normally in other diseases. The KNN and SVM classifier yielded similar results as the RF classifier (Figures 8A–F). By comparing the AUC value of three classifiers: RF(AUC:0.91), KNN (AUC:0.89), SVM(AUC:0.93), we can find SVM classifier behaviors best. To confirm the results above, MCC was implemented to select the optimal classifier to use in clinical applications. As illustrated in Figure 9, SVM had the highest MCC all the time, which represents high recognition accuracy and precision. To sum up, the SVM classifier has the best precision of SSR1 in PD.

## Discussion

Studies on PD biomarkers based on the GEO datasets have mostly used the peripheral-blood datasets (Wang et al., 2017, 2019; Wu et al., 2020; Yuan et al., 2020). Biomarkers corresponding to PD molecular neuropathological characteristics based on its pathogenesis can not only predict PD at an early stage but also assess the condition of PD patients and judge their prognosis. Therefore, it would b valuable to find biomarkers that are not only related to the pathogenesis of PD but also abnormally expressed in peripheral blood. This study is the first to combine brain tissue and peripheral-blood datasets to find potential biomarkers of PD. We used WGCNA to select five hub genes and constructed a PPI network through GEO data analysis to find six key genes that are abnormally expressed in the brain tissue of PD patients. We selected the three upregulated genes shared by the two analytical methods for further study. Since the ultimate goal was to find peripheral-blood markers, we verified the expression of the three hub genes in the peripheral-blood datasets. This combined with survival analysis showed that all three hub genes were significantly upregulated and were associated with the overall survival of patients. Through bioinformatics analysis, we further confirmed the applicability of the hub genes in animal models, which suggests they can be useful in the clinic. Through qPCR verification, we successfully reproduced the SSR1 disorders in the mouse SN, which was consistent with the bioinformatic analysis. However, GTF2H5 and RNF130 were verified in only one model, and we were unable to verify their value in both models, so we will not further study them in PD.

From the loss of dopamine neurons and the time curve of SSR1 brain expression, the imbalance of SSR1 expression is closely related to the loss of dopamine neurons. The more dopamine neurons are lost, the higher the expression of SSR1. Although we have not fully proven that SSR1 is involved in the damage to TH neurons, our experimental results do show that SSR1 is highly correlated with the damage to TH neurons and may indicate the severity of TH damage. Our results also show that when TH neuronal damage was below 20%, SSR1 expression was maintained to a certain degree. This suggests that the expression of SSR1 may be the response of glial cells to TH neuron damage. We also compared the behavioral curve with the curve of SSR1 expression in peripheral blood. SSR1 was upregulated in the early PD model or even when there is no obvious abnormality in behavior. SSR1 showed a certain degree of predictive power for PD in animal models.

The signal sequence receptor subunit (SSR) is a glycosylated endoplasmic reticulum (ER) membrane receptor associated with protein translocation across the ER membrane. The SSR consists of two subunits, one of which is SSR1. The main function of the endoplasmic reticulum is the synthesis and folding of secretory proteins. Changes in ER function will increase oxidative stress or protein N-glycosylation dysfunction, leading to the accumulation of misfolded proteins in the ER and triggering ER stress. Through KEGG analysis, we found that SSR1 was involved in ER stress. In a recent model of ER stress, it was found that long-term endoplasmic reticulum stress can induce the upregulation of mRNA encoding TARPa, namely, SSR1 (Nguyen et al., 2018). However, the significance of SSR1 in the PD model has never been confirmed. The impact of ER stress in PD has been a concern in recent years. It was first discovered in the PD model induced by MPP + rotenone. Long-term ER stress participates in the unfolded protein response (UPR) through high expression of genes involved in the pathological process of PD (Ryu et al., 2002). UPR-related signaling pathways are an adaptive cellular mechanism designed to restore ER homeostasis. Misfolded proteins can activate it to limit ER stress (Hetz et al., 2011). The activation of the UPR is controlled by the PERK, IRE1α, and ATF6 receptors on the ER membrane. Under normal circumstances, BiP binds to related receptors to inhibit its phosphorylation and the activation of downstream pathways. Under pathological conditions, α-synuclein directly interacts with BiP to trigger the phosphorylation of BiP, promote the dissociation of BiP from related receptors, and activate the UPR (Cooper et al., 2006; Jiang et al., 2010; Bellucci et al., 2011), thereby inducing downstream activation of the PERK axis, the IRE1α-XBP1 axis, and the EIF2α axis (Prell et al., 2012). Autopsy analysis of Parkinson’s patients has found that compared with the control group, patients with PD showed more phosphorylated PERK in the SN dopaminergic neurons. eIF2α, phosphorylated PERK, and α-synuclein coexist in the dopaminergic neurons of PD patients (Hoozemans et al., 2007), which further suggests that α-synuclein and long-term ER stress in PD patients are closely linked. The ER stress induced by tunicamycin can also lead to the accumulation of oligomeric α-synuclein (Jiang et al., 2010), indicating that the ER stress may also reversely aggravate the aggregation and toxicity of α-syn, forming a vicious cycle and exacerbating PD deterioration. We speculate that SSR1 may be a UPR-related mRNA that reflects the degree of ER stress. In the early stage of injury, abnormally aggregated α-synuclein activates the UPR to promote the upregulation of the SSR1 gene by binding to BiP to relieve acute ER stress. Therefore, the compensatory effect of dopamine neuron damage is not obvious at this time. When the ER stress becomes chronic, it exacerbates the accumulation of oligomeric α-synuclein, and the compensatory effect of the UPR cannot counteract the increasing accumulation of abnormal α-synuclein, which further triggers inflammation. At this time, dopamine neurons are significantly reduced, and animal behavior is also significantly abnormal. The expression of SSR1 continues to be upregulated. α-Synuclein activates the PERK axis in astrocytes, and the regulation of the UPR by α-synuclein is not limited to neurons. Considering that astrocytes participate in a variety of brain functions and support neuronal activity, activation of the UPR in these cells by α-synuclein may lead to harmful consequences. This may explain why SSR1 is still highly expressed when the expression of TH neurons in late PD is extremely low. Therefore, SSR1, which has abnormal expression in the early stage of PD (before obvious movement disorders), can be used not only as an early marker but also as an effective indicator of the severity, progression, and prognosis of PD.

For the first time, we applied the timeline of an animal model to the verification and exploration of hub genes, instead of knocking out target genes in an organelle. Exploration in mice may also lay the foundation for the next step toward clinical application. The most commonly used machine learning includes SVM, KNN, RF, and ANN (Artificial Neural Network). Since ANN is a multivariate input, it has no way to predict only SSR1. So we choose the other three classifiers to analyze SSR1 temporarily. Based on our analysis, we selected SVM to construct a computer model for clinical prediction. The application of artificial intelligence to the medical industry has gradually progressed, especially in the fields of early PD prediction and severity prediction (Zhan et al., 2018; Gupta et al., 2020). Recent advances in SVM have enabled the creation of computer models that can accurately perform many tasks involving prognosis of the disease and early diagnosis (Kaya, 2019). SVM has identified PD patients’ dopaminergic imaging markers (Prashanth et al., 2016), walking protocols (Rehman et al., 2019) and idiopathic REM sleep behavior disorder (Christensen et al., 2014) for early prediction. In this study, we established a SVM classifier model by identifying the peripheral-blood data of different samples that were from healthy or PD patients and continuously consolidated and improved the accuracy of the model through continuous calculation and screening of the data. In the future, as the number of clinical data samples increases, we can further improve the training results.

In future studies, we would like to further investigate whether the abnormal expression of SSR1 in PD patients is dominated by dopaminergic neurons or astrocytes. We also plan to study the possible mechanisms within cells. To improve the accuracy and sensitivity of diagnosis, the combination of neuroimaging and peripheral-blood biomarkers can provide better discrimination between parkinsonisms. The SVM can combine peripheral-blood data and images and differentially weight the two kinds of data to form an accurate judgment classifier model. This method is easily accessible and clinically applicable. It provides opportunities to develop an early diagnostic tool for PD patients, helping to save their dopaminergic neurons as early as possible.

## Data Availability Statement

The datasets presented in this study can be found in online repositories. The names of the repository/repositories and accession number(s) can be found in the article/Supplementary Material.

## Ethics Statement

The animal study was reviewed and approved by Animal experiment ethics committee of Nantong University. Written informed consent was obtained from the individual(s) for the publication of any potentially identifiable images or data included in this article.

## Author Contributions

MC and QZ designed the experiments. WZ and YW performed the experiments. WZ, YW, KC, and JS analyzed the data. MC and JS contributed to reagents, materials, and analysis tools. WZ and QZ wrote the article. All authors contributed to the article and approved the submitted version.

## Conflict of Interest

The authors declare that the research was conducted in the absence of any commercial or financial relationships that could be construed as a potential conflict of interest.

## Publisher’s Note

All claims expressed in this article are solely those of the authors and do not necessarily represent those of their affiliated organizations, or those of the publisher, the editors and the reviewers. Any product that may be evaluated in this article, or claim that may be made by its manufacturer, is not guaranteed or endorsed by the publisher.

## Footnotes

^1^ http://www.ncbi.nlm.nih.gov/geo

^2^ https://www.ncbi.nlm.nih.gov/geo/geo2r/

^3^ http://string-db.org

^4^ http://www.cbioportal.org/

^5^ http://david.ncifcrf.gov
