# Supplementary figures and images for: Blood SSR1: A Possible Biomarker for Early Prediction of Parkinson’s Disease
Source: Front Mol Neurosci. 2022 Mar 2;15:762544. doi: 10.3389/fnmol.2022.762544 (PMC8924528; doi:10.3389/fnmol.2022.762544)

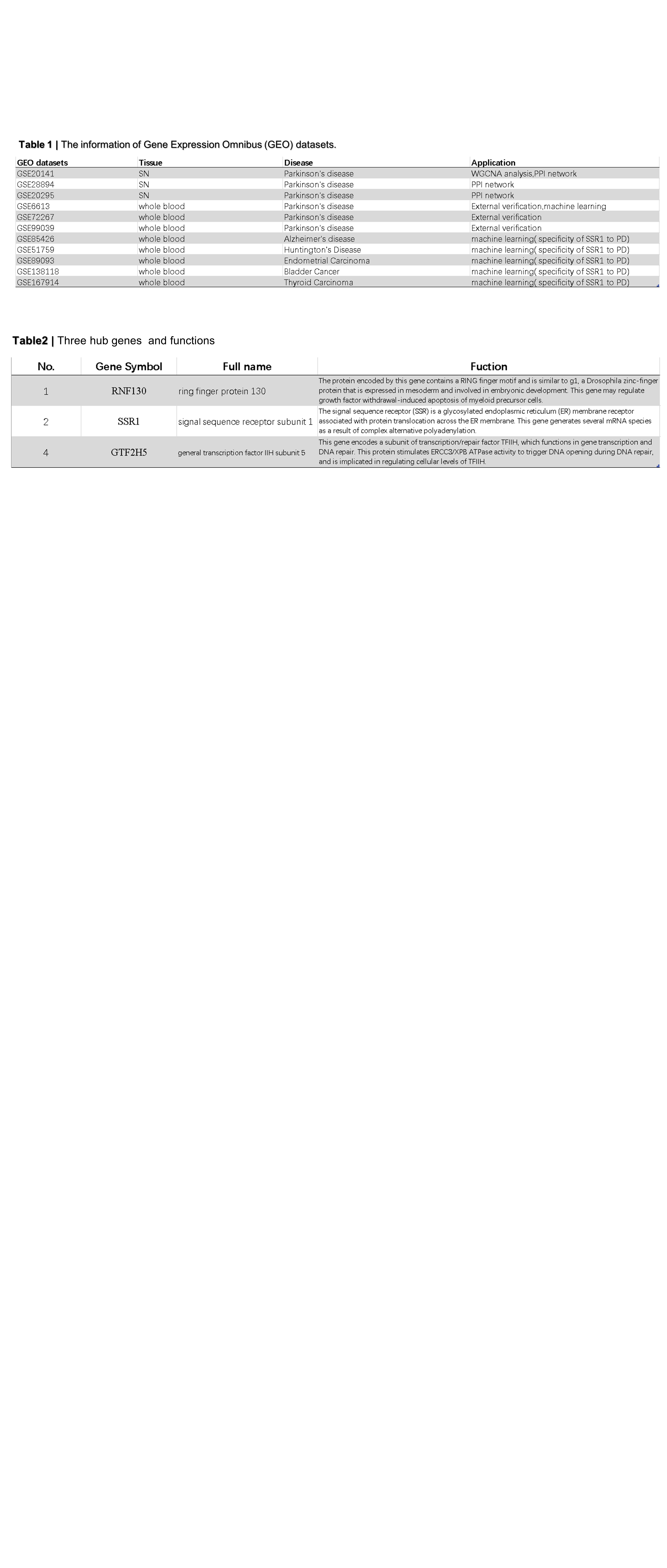

Supplement: Supplementary file 1 [file Image_1.TIF]
